# A qualitative exploration of older people’s lived experiences of homelessness and memory problems – stakeholder perspectives

**DOI:** 10.1186/s12877-023-04250-0

**Published:** 2023-09-12

**Authors:** Penny Rapaport, Garrett Kidd, Rosario Espinoza Jeraldo, Ava Mason, Martin Knapp, Jill Manthorpe, Caroline Shulman, Gill Livingston

**Affiliations:** 1 UCL Department of Mental Health of Older People, Division of Psychiatry, Wing B, Floor 6 Maple House, 149 Tottenham Court Rd, London, W1T 7NF UK; 2 Pathway, London, UK; 3 Care Policy and Evaluation Centre, London School of Economics and Political Science, London, UK; 4 NIHR Policy Research Unit in Health and Social Care Workforce, King’s College London, London, UK; 5 NIHR Applied Research Collaboration (ARC) South London, London, UK; 6 Healthy London Partnership, London, UK; 7 Marie Curie Palliative Care Research Department, Division of Psychiatry, UCL, London, UK; 8 Camden and Islington NHS Foundation Trust, London, UK

**Keywords:** Memory problems, Ageing, Homelessness, Inclusion health, Qualitative

## Abstract

**Background:**

The numbers of older people experiencing both homelessness and memory problems are growing, yet their complex health, housing and care needs remain undelineated and unmet. There is a critical gap in understanding what can improve the care, support and experiences of this group. In this qualitative study we explore how stakeholders understand memory problems among older people in the context of homelessness and consider what they judge gets in the way of achieving positive outcomes.

**Method:**

We conducted reflexive thematic analysis of qualitative interviews (n = 49) using a semi-structured topic guide, with 17 older people (aged ≥ 50 years) experiencing memory problems and homelessness, 15 hostel staff and managers, and 17 health, housing and social care practitioners. We recruited participants from six homelessness hostels, one specialist care home and National Health and Local Authority Services in England.

**Results:**

We identified four overarching themes. The population is not taken seriously; multiple causes are hard to disentangle; risk of exploitation and vulnerability; and (dis)connection and social isolation. The transience and lack of stability associated with homelessness intensified the disorienting nature of memory and cognitive impairment, and those providing direct and indirect support required flexibility and persistence, with staff moving beyond traditional roles to advocate, provide care and safeguard individuals. Memory problems were perceived by frontline staff and older people to be overlooked, misinterpreted, and misattributed as being caused by alcohol use, resulting in pervasive barriers to achieving positive and desired outcomes.

**Conclusions:**

Efforts to meet the needs of older people living with memory problems and experiencing homelessness and future interventions must reflect the complexity of their lives, often in the context of long-term alcohol use and current service provision and we make suggestions as to what could be done to improve the situation.

**Supplementary Information:**

The online version contains supplementary material available at 10.1186/s12877-023-04250-0.

## Introduction

Although definitions and criteria for measurement vary, the population experiencing homelessness is ageing in the United Kingdom (UK) and globally [1, 2] with numbers of people experiencing homelessness aged over 65 years predicted to triple by 2030 in the United States (US) [3]. In their definition, the European Typology of Homelessness and housing exclusion (ETHOS) launched in 2005, incorporates rooflessness (without a shelter of any kind, sleeping rough), houselessness (with a place to sleep but temporary in institutions or shelter) as well as those living in temporary, insecure, or inadequate housing [4]. In this study we have adopted this approach with a specific focus on those experiencing houselessness and living in temporary hostel accommodations or shelters.

Those experiencing homelessness have higher levels of cognitive impairment than those of equivalent age who have not [5–9], and are significantly more likely to have Alzheimer’s disease and related dementias than stably housed populations [10, 11]. People experiencing homelessness frequently have long-standing and interacting physical and mental health conditions, substance misuse, and history of traumatic brain injury (TBI) [12–14]. This can lead to accelerated ageing [8, 15–17], associated with increased service use and early death [7, 10]. Older people experiencing homelessness have multiple causes of memory disorder, not all of which are classified as dementia, and diagnosis is challenging [7, 18]. It can be difficult both in research and clinical contexts to disentangle these co-occurring factors, especially if relying on self-reporting of symptoms and there are often no relatives or friends available to give more detail. The nature of homelessness can mean that people move areas and are not able to be followed up by a team for the diagnostic process. Additionally, those experiencing homelessness face multiple and entrenched forms of social exclusion which mean that they are likely to face barriers to timely and accurate diagnosis within existing health services structures (7, 19). Barriers may include services having zero tolerance for drugs and alcohol, strict inclusion criteria, and those experiencing homelessness being less likely to have family and friends to support and advocate for their needs [19]. However, many present with memory and other cognitive problems impacting everyday functioning, vulnerability, disinhibition and complex health, social and housing needs. Here we use the term ‘memory problems’ to include the population of older people experiencing homelessness, presenting with dementia-like symptoms, often without any formal diagnosis.

Research to date has focused on prevalence, risk factors and assessment of memory problems in older people experiencing homelessness. Little is known about how frontline staff support people with such needs and how people with memory problems experience homelessness. In the UK, Manthorpe et al. identified challenges within care pathways including lack of flexibility in dementia support services and lack of appropriate housing [20]. In England, hostels are commissioned to provide short-term accommodation (usually with a 2-year maximum stay, often far shorter) with a remit to move people into less supported environments and a focus on recovery. Recovery is a contested and heterogenous term which dominates in homelessness services. Approaches commonly promote autonomy, self-management, meaningful activity and reclaiming preferred identities [21]. In practice, however, this often translates to approaches that focus on getting individuals to live more independently with lower levels of support. Such approaches have been critiqued for leading to further exclusion of those with the highest or most complicated levels of need [22]. Some accommodation is commissioned as ‘complex needs hostels’, specifically for those with additional physical, mental health or substance misuse needs, although hostels do not provide direct health and personal care input [20, 23]. Those experiencing homelessness often experience complex ill health and multimorbidity making it harder for them to move on from temporary accommodation [17, 23, 24] with high personal and social care needs and emergency and out-of-hours service use [10, 20]. This is compounded by the lack of suitable, tailored accommodation to move on to [20, 25].

There is a critical gap in understanding what could lead to improvements in care, support and housing and inform intervention for older people experiencing memory problems and homelessness. Although studies have explored the lived experiences of older people experiencing homelessness [26], we found only one Australian study which privileged the voices of people living with memory problems and experiencing homelessness, through in-depth qualitative interviews, and this was in a single hostel and did not focus on older people [27]. Qualitative and ethnographic studies are increasingly being conducted to inform practice, intervention development and policy in homelessness [19, 28]. We aimed to use qualitative interviews with stakeholders (people with memory problems experiencing homelessness, hostel staff and managers and health, housing and social care practitioners) to explore the experiences of those living with memory problems and experiencing homelessness as well as those supporting them. We considered what they perceive to be barriers to achieving positive outcomes for older people experiencing memory problems.

## Methods

### Setting, participants and procedures

In line with the Cochrane stakeholder engagement framework [29], we chose our stakeholder groups based upon our research questions, discussion with a range of stakeholders when planning the research, and experiences from other projects with similar objectives [19, 30], including ‘consumers and the public’, ‘practitioners and policy makers’ and ‘healthcare managers. We recruited hostel staff and managers working with older residents self-reporting memory problems in six complex needs hostels and one specialist care home in London run by five voluntary service providers. We selected services of varied size, supporting individuals (across the age range, varied physical and mental health needs), with differing levels of on-site support.

We recruited people experiencing homelessness with memory problems who were aged 50 or over who were now living in temporary or hostel accommodation. We excluded people who were currently rough sleeping as the immediate priority would be to support them into a place of safety. The decision to focus on those aged 50 and over was based on: (1) Existing evidence that people experiencing homelessness ≥ 50 have more comorbidities, cognitive problems and unmet needs than younger people [8, 31]; (2) The former UK Coalition on Older Homelessness definition of ‘older’ homeless people as those aged ≥ 50 years [1]; (3) Discussions with academic, policy and service providers. During this discussion, a common viewpoint was that this is an overlooked, vulnerable, and relatively invisible population with distinct needs from the younger homeless population. Additionally, there was a strong feeling that we should focus upon those living in temporary or hostel accommodation rather than those who were currently rough sleeping. In part this was because many will have previously been sleeping rough and could draw on this experience but from the relative safety of being in accommodation where at least their basic needs may be met. We excluded those lacking capacity to consent to participate as our experience is that they would likely struggle to participate in a qualitative interview due to the severity of their impairment. Mental capacity to give informed consent was initially judged by experienced hostel staff who knew the participants best. This was followed by researchers undertaking a brief assessment of the participants capacity to give informed consent as part of the consenting procedure.

We accessed health and social care practitioners (HSCP) and managers involved in commissioning or provision of support for older people experiencing homelessness and memory problems via existing networks, from participating voluntary organisations and NHS and local authority (LA) services in London, Sussex and Yorkshire.

**Fig. 1:**
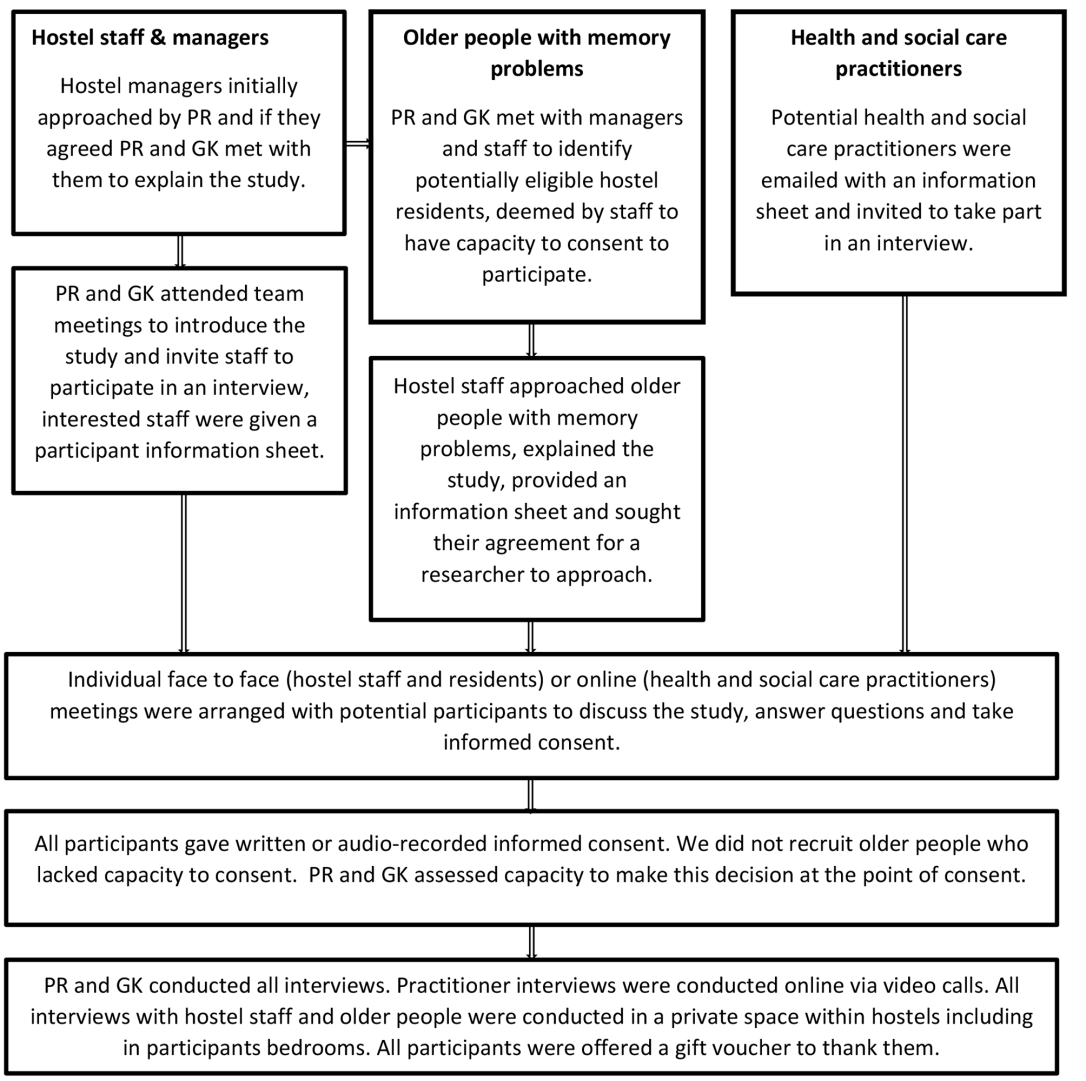
Approach, recruitment and data collection procedures

We purposively selected participants to ensure we interviewed people of different genders and ages, ethnicities, nationalities, roles and work experiences (professionals), self-reported physical health status, care needs, substance use (older people). This information was collected via background and demographics questionnaires prior to conducting the recorded interviews. We used a semi-structured interview schedule based on the literature and research team expertise with different versions for older people, hostel staff and managers and health and social care practitioners. PR and GK met weekly to review recruitment and reflect on initial themes. We ceased interviews after reaching thematic sufficiency, the point at which no further themes emerged from reflections on additional interviews [32]. Interviews were audio recorded, entered into NVivo 12 software, transcribed verbatim and anonymised. Recruitment and data collection procedures are detailed in Fig. 1 and semi-structured interview schedules can be found in Appendix A.

### Data analysis

We took a reflexive thematic analytic approach [33] informed by a ‘contextualist’ or ‘critical realist’ position which neither denies the impact of social context upon people’s experiences nor overlooks their material or lived experiences [34, 35]. This approach is particularly fitting in research with those experiencing homelessness where day-to-day experiences are undeniably framed by institutional culture and social context but where the uniqueness of lived experiences can be easily overlooked. We also drew upon the background information collected at the time of interviews to contextualise our sample. PR, GK, AM and REJ independently and systematically coded each transcript into meaningful fragments labelling initial codes. 25% of the transcripts were coded by two researchers who met regularly to discuss initial themes corresponding to our research questions and to resolve any discrepancies. We maintained reflective diaries and met regularly to share our experiences [36]. PR then organised the data into themes exploring commonalities and differences between different perspectives, revising iteratively. We discussed initial findings in the wider research team (MK, GL, JM and CS) and individuals with lived experiences of homelessness who were not participants in the study, to enhance the credibility and dependability of our findings [37]. Following discussion with these individuals we revisited our themes and the language we had used to label quotations, attempted to integrate their reflections, refining our thematic analysis whilst keeping the voices of participants at the centre of our analysis. We present a checklist of methods used against the Standards for Reporting Qualitative Research [38] in Appendix B.

### Ethical considerations

London (Brighton and Sussex) National Research Ethics Service approved the study (reference: 21/LO/0541) on 6th September 2021. This study was performed in accordance with the declaration of Helsinki and research was conducted in accordance with the protocol approved by the ethics committee. All participants gave written or verbally recorded informed consent prior to interviews. All participants had capacity to consent to participate. We followed our established safeguarding procedures and worked in line with the hostel processes regarding any potential safeguarding issues that were observed or disclosed to research staff.

## Results

### Study participants

Between September and December 2021, we interviewed 17 older people who self-identified as having memory problems, 17 HSCP and 15 hostel staff and managers. We recruited hostel staff and older people from six complex needs hostels (sizes ranged from 30 to 79 beds); two of these provided accommodation to men only, three of these were for older people only and one hostel had an on-site care agency providing additional care to residents. We also interviewed staff and residents in a specialist Care Quality Commission (CQC) registered care home for men aged over 50 who had been homeless and had substance misuse problems (29 beds). None of the participants with memory problems were aware of a diagnosis of dementia. HSCP were recruited from inclusion health services (supporting those at risk of multiple social exclusion) (n = 5), homelessness service providers (n = 4), LA social care (n = 2), NHS secondary care memory services (n = 2), substance misuse services (n = 2), older people’s inpatient services (n = 1) and a LA housing department (n = 1). Participant characteristics are presented in Tables 1, 2 and 3.

**Table 1:** Characteristics of people experiencing homelessness and memory problems

Characteristic	Category	*n* (%) or mean (*SD*)
Age		62.3 (8.7)
Gender	Female Male	1 (5.8) 16 (94.2)
Ethnicity	White British White Irish / Welsh Black British Black other	11 (64.8) 3 (17.6) 2 (11.8) 1 (5.8)
Accommodation	Hostel Care home Independent flat on hostel site	16 (94.2) 1 (5.8) 1 (5.8)
Time in current accommodation	< 2 years 2–3 years 4–5 years 6–10 years 11–15 years Unknown	6 (35.4) 3 (17.6) 2 (11.8) 3 (17.6) 2 (11.8) 1 (5.8)
Contact with family	Yes No	7 (41.1) 10 (58.9)
Has seen a doctor about memory problems	Yes No Unsure Did not specify	6 (35.4) 5 (29.4) 4 (23.4) 2 (11.8)
Current physical health problems	Yes No	15 (88.2) 2 (11.8)
Current mental health problems	Yes No	12 (70.6) 5 (29.4)
History of brain injury	Yes No	15 (88.2) 2 (11.8)
Current alcohol use	Monthly or less 2–4 times/month 2–3 times/week 4–5 times/week 6–7 times/week	2 (11.8) 2 (11.8) 2 (11.8) 3 (17.6) 8 (47.0)
History of heavy alcohol use	Yes No	12 (70.6) 5 (29.4)
Current (illicit) drug use	Never 2–3 times/week 4–5 times/week 6–7 times/week Did not specify	11(64.8) 2 (11.8) 1 (5.8) 1 (5.8) 2 (11.8)
History of heavy (illicit) drug use	Yes No	10 (58.9) 7 (41.1)

**Table 2:** Characteristics of Hostel staff and managers

Characteristic	Category	*n* (%) or mean (*SD*)
Age		42.2 (10.4)
Gender	Female Male	10 (66.7) 5 (33.3)
Ethnicity	White British Asian British White other Black British	11 (73.3) 2 (13.3) 1 (6.7) 1 (6.7)
Job title	Managers/deputy managers/team leaders Complex needs/health/specialist workers Support workers Project workers	4 (26.6) 4 (26.6) 4 (26.6) 3 (20.0)
Time working in current role	< 1 year 1–3 years 3–5 years 5–10 years 10 + years	3 (20.0) 7 (46.6) 3 (20.0) 1 (6.7) 1 (6.7)
Time working in homelessness sector	< 1 year 1–3 years 3–5 years 5–10 years 10 + years Did not say	3 (20.0) 2 (13.3) 2 (13.3) 5 (33.3) 2 (13.3) 1 (6.7)
Past experience working with people with memory problems	Yes No Did not specify	11 (73.3) 3 (20.0) 1 (6.7)

**Table 3:** Characteristics of Health and Social Care Practitioners

Characteristic	Category	*n* (%) or mean (*SD*)
Age		42.2 (10.4)
Gender	Female Male	10 (58.8) 7 (41.2)
Ethnicity	White British White other Asian other	15 (88.2) 1 (5.9) 1 (5.9)
Professional role	Specialist nurse Service manager Psychiatrist Psychologist Coach Geriatrician GP Housing commissioner Social worker Speech and language therapist	3 (17.6) 3 (17.6) 3 (17.6) 2 (11.8) 1 (5.9) 1 (5.9) 1 (5.9) 1 (5.9) 1 (5.9) 1 (5.9)
Time working with memory problems	1–3 years 3–5 years 5–10 years 10 + years	1 (5.9) 2 (11.8) 3 (17.6) 11 (64.7)
Time working in homelessness	< 1 year 1–3 years 3–5 years 5–10 years 10 + years	3 (17.6) 1 (5.8) 1 (5.8) 3 (17.6) 9 (52.9)

### Qualitative findings

We identified four themes common across participants living with memory problems and experiencing homelessness, HSCP and hostel staff and managers. These were (1) The population is not taken seriously; (2) Multiple causes are hard to disentangle; (3) Risk of exploitation and vulnerability and (4) (Dis)connection and social isolation (see Table 4).

**Table 4:** Table of themes and differences between stakeholder groups

Overarching theme	Subtheme	Stakeholder contribution			
		**PWMP**	**HSCP**	**HW**	
The population is not taken seriously	Feeling overlooked and dismissed	x	x	x	
	Staff advocate and persist		x	x	
Multiple causes are hard to disentangle	Filling in gaps: Missing narratives	x	x	x	
	Existing pathways do not fit		x	x	
Risk of exploitation and vulnerability	Feeling unsafe: Questioning one’s perceptions	x		x	
	Disorientation increases risk	x	x	x	
(Dis)connection and social isolation	Self-reliance and resilience	x		x	
	Meaningful interaction and identity outside homelessness	x	x	x	

Notes: HSCP = Health and social care practitioners, PWMP = Person with memory problems, HW = Hostel worker

#### The population is not taken seriously

Stakeholders viewed older people with memory problems as easily dismissed at societal and individual levels, with their complex needs unmet by health, social care or housing services. Practitioners and hostel staff highlighted direct consequences upon health outcomes for older people. These related to not being offered appropriate investigation or intervention, including medication. Examples given included leg ulcers not being treated effectively, fractures not being identified following a fall or underlying neurological and gastrointestinal conditions being misdiagnosed or attributed to alcohol use. Those experiencing memory problems described the emotional impact of being dismissed or overlooked. Participants felt that older people were blamed for their symptoms or behaviour:

Automatically their behaviour will be attributed to that situation and that’s it then, they are bad, they are naughty and it’s like, have you understood what’s going on for them that day, have you understood the frustration. So, without finding out what’s happened to them – fundamental over-attribution error. (Practitioner 1)

Stakeholders connected this to pervasive negative assumptions held about drug and alcohol use, with frontline staff themselves often not feeling listened to or taken seriously:

I think it happens quite a lot with the drug users immediately when I go: ‘he has a bad memory’, ‘well he’s a drug user’. I mean, they just write it off as drug use when actually, sometimes it’s not drug use… Whenever I go to the doctors with him, I am just ignored whatever we want to say. (Support worker 1)

There was a common perception that memory and other cognitive difficulties were overlooked or underestimated and therefore care and support were missing or inappropriate:

We had loads of safeguarding [concerns about adult abuse], huge amounts of evidence over a long period of time and the Care Act assessor said, “we’ve got absolutely no concerns about this person’s capacity and cognition”. (Physician 1)

Additionally, all stakeholder groups referred to difficulties with understanding and processing being misattributed as behavioural rather than indicative of an underlying deficit and therefore attempted solutions and interventions failed:

When we’re not certain what their understanding was… So, for example, if somebody has been issued with a notice to quit [pending eviction] and then a behaviour agreement do they understand what’s in the behaviour agreement, so can they therefore meet that agreement or are we just going to set them in to be evicted? (Allied health practitioner 1)

At times, staff perhaps overestimated individual’s abilities to recognise difficulties, attributing a level of personal responsibility to those they were supporting. This was discussed by the older participants:

Just be patient with people. Especially with us older ones who really forget things, yeah? Not just people that are putting things off because they don’t want to face realities, yeah? So, I think staff actually think that sometimes, yeah? But in fact, a lot of people are quite genuinely… Have proper memory problems. (Hostel resident 1)

#### Feeling overlooked and dismissed

Participants with memory problems felt distressed when they felt disrespected or dismissed by strangers or professionals, especially when they sought assistance with functional difficulties in public, such as forgetting a PIN (personal identification number for bank) or getting lost. Additionally, they felt that difficulties resulting from memory loss were often minimised or ignored by hostel staff:

‘Well, you packed your things so you must know’ that is it. It is the total whole sum. ‘Oh, you have got something missing, where is it gone? Oh, you probably lost it in the move or left it in this place or that place’. (Care home resident)

Several individuals recounted how they felt embarrassed and frustrated, leading them to express anger towards hostel staff, occasionally resulting in threat of or actual eviction. Staff articulated a personal dilemma as well as a challenge for mainstream older people’s services, that older men experiencing homelessness and memory problems did not fit with how older people are expected to behave, often being labelled as anti-social, resulting in withholding of support or exclusion from services:

Often, they’ve had a history of much more chaotic drug and alcohol use, sometimes still using despite their cognitive deficits and a lot of places, they just won’t touch people with a barge pole to be honest. (Substance misuse psychiatrist)

#### Staff advocate and persist

Practitioners and frontline staff and managers expressed exasperation that they had to constantly fight for those they were supporting:

I mean it is several places above my pay grade, but at least I know now who to go to start to try and unblock these issues… It is my job not to let things go. (Physician 1)

He was continuously exploited. Trying to support him to meet his ongoing needs, trying to get him to appointments. We just worked tirelessly. (Nurse 1)

They contrasted this to the care of other older people not experiencing multiple disadvantages, and so felt an added sense of responsibility:

At least in kind of mainstream older care you often have next-of- kin or family who can advocate for people and at least tell you about what they think they might have wanted. (Physician 2)

This was illustrated in the account of a hostel worker who described how one resident had moved from the hostel to a care home only to be evicted back to the hostel:

Since they come here, we start like fighting for their case. And then we managed to get them to move to a care home. All good, fine, but then they move from here… if they’re not supported properly or they don’t have anyone to advocate for them [they get evicted] they get lost. (Hostel team leader 1)

#### Multiple causes are hard to disentangle

Understanding was framed in terms of complexity, multiple transitions and traumatic events, common for this group. This resulted in lack of clarity around the cause and nature of memory and other cognitive problems, and difficulty conducting accurate and meaningful assessment and diagnosis:

I mean, you know, normally you wherever I’ve been involved before, it’s like kind of you’ve got the mental health issues going on separate to people drinking, smoking, substance misuse, separate to people with memory problems. But here it’s all under one umbrella. (Project worker 2)

Yeah, I again I can’t or won’t be able to say, like you know you know these things that you say there could be a million and one reasons for behaviours or their presentation. (Allied health practitioner 2)

Older participants related memory difficulties to traumatic experiences, often stating that alcohol use and head injury seemed to have caused memory difficulties:

I think it’s just my way of living. I’ve been on the streets for a long time. I’ve been battered about a lot. (Hostel resident 1)

#### Filling in gaps: missing narratives

Older people’s narratives were fragmented; heightened by multiple transitions and social disconnection in the context of broken ties with family and geographical moves. They reflected on how they could not piece together recent or more distant history, resulting in confusion, intensified by the transitory nature of their lives in temporary accommodation:

I don’t know what I am doing in this place, I really don’t. They just stuck me in this mad house…I don’t know…This place is changing my brain. (Hostel resident 2)

The timeline of events that these people have gone through, you know, right [resident] is 50, nobody could process that and now they don’t remember themselves. (Nurse 2)

Frontline staff were expected to provide information that they did not have, working hard to fill in the gaps, themselves acting as ‘the eyes and ears really’ (Complex needs worker 1) for hostel residents. Practitioners and hostel staff described the need for ‘digging and a lot of unpacking’ (Allied health practitioner 3).

#### Existing pathways do not fit

Practitioners, commissioners and frontline staff were clear that the multimorbidity and complex presentation of older people experiencing memory problems interacted with fragmented clinical pathways, further excluding this group from accessing support:

When the cognitive impairment is due to substances of or a chronic mental health issue like schizophrenia or something like that, it is not entirely clear who is commissioned, I mean, rather let’s be clear, there is no one commissioned to look after these people and their cognitive difficulties. (Psychiatrist 1)

Practitioners expressed frustration about inflexibility in services:

People who perhaps have more difficulties and cognitive impairment fall through the cracks and are unable to, on their own, navigate their way through an increasingly fractured and complicated patchwork of services. (Psychiatrist 2)

For frontline staff this was experienced as helplessness, inability to resolve issues and feeling that their concerns were not listened to or acknowledged:

We feel detached because we won’t be let known along the process as to what’s happening. Is something been accepted? Are they actually going to get in anywhere? (Specialist health worker 1)

Ultimately there was a sense of individuals bouncing between services and pathways, also apparent in older participants’ accounts when they expressed confusion and frustration about their experiences.

#### Risk of exploitation and vulnerability

Extreme vulnerability faced by older people experiencing memory problems and homelessness pervaded accounts, often framed as interaction between people’s living arrangements and cognitive and functional impairments. Older people gave stark accounts of feeling unsafe:

It is a living nightmare, constantly people who ask me for money and they borrow money from me and knocking at your door and early hours of the morning for a bit of tobacco, you know… Anyone could just knock the door and just push themselves through the door or whatever. And that’s why I don’t trust anyone in here. (Hostel resident 3)

Staff discussed the challenges of keeping people safe, feeling that safeguarding processes and legal frameworks supposed to protect vulnerable individuals and their rights, were at odds with living in hostels:

Like we can’t just say, oh, you can’t leave the building. Of course, they can leave as they please. So that’s definitely a problem, when they go out because when they are on the street, they can get robbed, they can fall, they can forget how to get back. (Hostel team leader 1)

#### Feeling unsafe – questioning one’s perceptions

Older people interviewed often doubted their own perceptions, worrying that others were intentionally taking advantage of their memory difficulties. Sometimes this was accompanied by uncertainty about what had been happening:

It makes you untrusting. Yeah, because a certain something goes missing or you’ve put something down and you think you’ve put it in one place. When you looked there, it’s not there, yeah? You start thinking, “Who’s been in my room?”, yeah? “I was sitting here. Did I see them go towards it?” You do. It makes you paranoid, yeah? That’s what’s going on in your head, yeah? You forget that you actually forget things, yeah? (Hostel resident 1)

At times the frustration ignites within yourself. People try and use it against you, that gets me annoyed. At first I thought I was paranoid, then I realised people do enjoy mucking up the next person, I think they are sick individuals. (Hostel resident 4)

Frontline staff felt that this ‘paranoia’ was warranted as other residents in hostels sometimes did target more vulnerable residents but acknowledged that some individuals forgot or misperceived what had happened. Staff worked hard to minimise this uncertainty:

We looked at GPS tracking devices, stuff like that. It can be very challenging though, because it disorientates him and confuses him, gives him sense of paranoia, and general confusion. So they may not recognize the hostel staff - when [care workers] come in he might not recognize them and we have to sort it. (Complex needs worker 2)

#### Disorientation increases risk

Getting lost and disoriented commonly resulted in risky situations for older people. Practitioners and hostel staff felt that individuals were especially likely to go missing when discharged from hospital if cognitive difficulties were missed by staff or individuals were deemed to have capacity to self-discharge:

That’s basically when he goes missing, when he leaves the hospital. I mean, like with [name], he was missing like three or four days, and I found him after four days while I was going back home. He was sitting on a bench, and it was freezing. So, I took him back here and he couldn’t get up for the first couple of minutes because he was all stiff. (Hostel team leader 1)

Older people themselves described getting lost and forgetting where they were staying as frightening, frustrating and at times embarrassing:

Well, you just feel upset sort of thing, even though you are embarrassed, you just be like crying sort of thing, like you know where you want to get to, you know, you can kind of find the tube station. (Hostel resident 5)

#### (Dis)connection and social isolation

Older people experiencing homelessness and memory problems spoke of profound isolation and disconnection especially in hostels. They highlighted how their social world was diminished, contrasting this to their younger, socially connected selves. They related this to fears of being exploited or attacked, embarrassment and sometimes to feeling physically frail or unable to engage in conversation or activity outside of their bedrooms:

You feel vulnerable sometimes because, you know, sometimes you say hello to somebody, and somebody takes offense to that… I keep a low profile here. (Hostel resident 6)

Yes, you go to have a conversation and the words come but then all of a sudden they dislodge, and then your confidence goes. (Hostel resident 7)

Staff suggested that this isolation, although not unique to residents was more extreme and detrimental in those experiencing memory problems:

If and when somebody has an issue of memory, they are insecure and they don’t have that confidence they will try to shut themselves behind the closed door most of the time and won’t socialize with other people as much. And that makes them vulnerable. (Project worker 1)

And I think with memory problems that isolation just makes it worse. And, you know, can accelerate its deterioration. (Allied health practitioner 4)

#### Self-reliance and resilience

Older people reflected that a slower pace of life suited them, contrasting it with the more complicated relationships and interactions from the past. They framed self-isolation as self-reliance and independence; feeling that they had autonomy and freedom over their daily lives:

Support at the end of the day, I support myself, and that’s it, I don’t rely on anyone. I’m not even relying on the key worker. (Hostel resident 3)

I don’t, I don’t like back seat drivers… through this world, through this crazy world, I want to be the driver, I want to be the one behind the wheel, does that make sense to you? (Hostel resident 8)

Staff faced a dilemma as to how much to support those with memory problems, offering support at a distance, but sometimes feeling unsure about intervening:

I want to be able to help them in the way he would want. He’s like, he’s very independent, so it’s like when I meet him, it’s like he doesn’t need me. So, when I try and get information from him, it can be quite difficult and he just kind of just like, ‘I’m fine, can I go?’ (Support worker 2).

#### Meaningful interaction and identity outside homelessness

Hostel residents spoke of interacting little outside of their bedrooms other than about alcohol use or practical matters with staff. All stakeholders felt this worsened during the COVID 19 pandemic but that this was not the only factor:

They used to have a lunch club for all the oldies down there. It was great, that sort of thing. You know, for people with memory problems where they are safe, and they can interact with others. (Team leader 2)

A good outcome for me is somebody who has an identity away from homelessness, their preferred identity and it’s literally like helping connect people and creating opportunities to make that possible. (Psychologist)

Although older people spoke about isolating themselves, this was not what they chose and they wanted access to more meaningful interactions:

You know, for a start, I want to be able to do ordinary day by day things… have the door open, know what I want, not to be ignored in here. (Hostel resident 9)

Hostels themselves were perceived as inherently untherapeutic, incompatible with positive outcomes for older people with memory problems, particularly those needing additional care and support:

Whenever I see residents in their rooms, it always looks like, you know, it’s always a really unpleasant environment, it’s just undignified and not good. (Physician 1)

It’s not a peaceful, homely kind of atmosphere, you know, it’s, it’s just a roof over your head, basically. (Hostel resident 6)

## Discussion

### Main findings

This is the first qualitative study to explore the perspective of those living with memory problems and experiencing homelessness, together with the perspective of those supporting them. In our analysis we consider how different stakeholder groups navigate and negotiate complex experiences in the context of current service provision in England. Reported negative assumptions concerning people experiencing homelessness, especially related to alcohol and substance use, cut across themes, with staff and older people feeling overlooked and dismissed. Barriers to support and stigma contributed to negative emotions, cognitions and behaviours experienced by older people, with real consequences such as eviction from hostels or care homes. O’Carroll (2019), in their ethnographic study of service use amongst those experiencing homelessness, argues that external barriers to access contribute to internalized barriers, including embarrassment, fear and presumptions of inaccessible services [28]. This aligns with our findings, where older people made their own fatalistic assumptions of inevitable, negative outcomes and the need for self-reliance, based upon earlier and often repeated negative experiences, alongside a frequently unsafe and potentially dangerous present.

Our analysis, informed by a critical realist perspective demonstrates how lived experience of homelessness in the context of memory problems is framed by a combination of complex, interacting factors at individual, relational, social/environmental and structural levels [39]. For example, our analysis highlights how although individuals may have multiple, intertwined causes for their difficulties which cannot simply be disentangled, services tend to be siloed and focus on one dimension of experiences such as ‘memory problems’, ‘substance misuse’ or ‘mental health’. Additionally, rather than being enabled or facilitated to minimise the impact of cognitive deficits, hostel environments further isolate and alienate those living with memory problems. Those experiencing memory problems are known to have worse health and social care outcomes [8, 20] with functioning deteriorating over time [24]. Our findings highlight how this complexity is experienced and addressed at both individual and systemic levels. This interacts with experiences of deep and multiple social exclusion homelessness [40], and the fragmentation and narrative disconnection central to older participants’ experiences. Studies exploring the lived experiences of stably housed individuals living with dementia have constructed the loss of meaningful relations with self and others and disorientation in space and place as ‘existential homelessness’ [41, 42]. Those living with dementia may be able to benefit from relational interdependence that facilitates living well and meaningfully and supports a coherent narrative [30, 43, 44]. This was strikingly absent for our participants, fuelling mistrust and paranoia and contributing to difficulties for staff working hard to fill gaps and make connections.

### Differences between stakeholder perspectives

We were struck by commonalities in perspectives across stakeholder groups, particularly regarding the challenges of living in hostel settings with memory problems and negative societal assumptions perceived to be held about older people experiencing homelessness and memory problems. There were some areas of divergence between health and social care practitioners and people living with memory problems. Those living with memory problems movingly described doubting their own experiences of losing or forgetting. Underlying this was mistrust of those around them, coupled with awareness of their memory or perceptions potentially failing them. Connected to this was a sense from those living with memory problems that ultimately, they needed to rely upon themselves, based on past experiences of having been let down by people and services [19, 28]. Health and social care practitioner accounts did not include awareness of these elements of lived experience, but hostel staff narratives were clear about it, and described working hard to support from a distance and maintain a balance between supporting independence and mitigating potential risk.

These differences have implications for wider policy and service planning, particularly related to those living with memory problems successfully moving on to settled accommodation where there is currently a gap in existing understanding and evidence of what works [45, 46]. The discrepancy in perspectives highlights the importance of approaches which include realistic levels of support without undermining people’s sense of autonomy. In the future, our findings may inform a more nuanced understanding of the needs of our target population and speak to the potential benefits of peer-support interventions with older people with memory problems in homelessness settings [47]. It is pertinent that hostel staff perspectives were aligned with both those of the older people and the health and social care practitioners interviewed. This suggests that they could and should play a central role in bridging and therefore supporting, successful transition and their expertise acknowledged and valued as a knowledgeable yet invisible ‘dementia care workforce’ [20].

### Clinical implications

Rehabilitation and recovery-based approaches, dominant in the homelessness sector, may be limited for this population. As noted above, recovery can be variously defined and interpreted, and approaches vary across mental health, substance misuse and homelessness services. In the context of this study, we are referring broadly to approaches that promote individuals moving towards living more independently with lower levels of support [21]. Such approaches have been critiqued for leading to further exclusion of those with the highest or most complicated levels of need [22], and a gap in evidence of what works in supporting people to move on successfully from hostel accommodation has been highlighted [45]. Additionally, dementia is a progressive and declining condition with no available disease modifying treatments, and although the effects of alcohol related brain damage (ARBD) may improve when people abstain from or reduce alcohol consumption, abstinence or significant reduction in alcohol consumption would rarely be seen in hostel environments. Alongside memory problems, executive dysfunction and behavioural and mood impairments are common amongst older people experiencing homelessness [11], however our findings suggest these may be widely missed or misinterpreted. Cognitive deficits reduce everyday functioning and unintentional behaviours are sometimes interpreted as non-cooperation, especially in the context of ongoing alcohol use. Service delivery for older people with cognitive impairment who are homeless may require targeted adaptation and in-reach health services to hostels [48]. Others advocate for tailored and intensive support for older people experiencing homelessness [1, 31], with those with memory problems particularly vulnerable [20]. More broadly, framing our findings related to older people with memory problems through the lens of ‘multiple exclusion homelessness’ reinforces the need for services and policies to reflect the complexity and intersectional nature of peoples’ experiences rather than offering unidimensional approaches which do not fit [22].

Our findings align with work focused on the care and support needs of those living with ARBD which has highlighted the need to challenge the stigma impacting upon those experiencing memory problems and homelessness in the context of alcohol use, as well as the need for bespoke housing and whole systems approaches to intervention [49, 50]. Approaches which are flexible and incorporate harm-minimisation approaches, reflect individual needs and preferences, account for complexity, and which are relationship focused, have been identified as effective components of interventions targeting substance misuse by people experiencing homelessness [51, 52]. Our findings provide evidence for the importance of in-reach provision to hostels and for specialist input for staff in hostels, recognizing their role supporting those living with memory problems until better supportive accommodation is made available.

We will use these findings, combined with results from ongoing ethnographic work and existing literature in inclusion health [46, 53] and dementia care staff support interventions [54, 55], and existing work in the areas of palliative care [19], substance misuse [51, 52] and ARBD [50] in the context of homelessness, in the coproduction of an intervention for hostel staff and managers. The intervention will focus on supporting staff to integrate changes to their practice with older people with memory problems. Based on our findings, and the voices of those interviewed, the coproduced intervention may include education on the multiple causes and impact of memory problems, including a focus on ARBD and dementia, understanding mental capacity, communication and managing distressed behaviours, promoting social interaction, and communicating with external agencies regarding memory and other cognitive problems.

### Strengths and limitations

This is a large qualitative study incorporating a range of stakeholder views in a largely unexplored area. In adopting a cross-sectional design we are capturing people’s experiences at one time point and We accessed a breadth of viewpoints, which contributed to the richness and relevance of the analysis [37] addressing a gap in existing literature [26]. This research was conducted entirely in England, predominantly in London areas with relatively high levels of homelessness and established services. We also recruited from voluntary or third sector organisations of varied size and resourcing. It is notable that even in relatively well-resourced areas with established services, profound challenges existed. In less well-resourced areas gaps and challenges may be greater, supporting the transferability of these findings. Our experience was that the majority of those eligible to take part in hostels were white men, reflective of the UK older hostel population more broadly [20, 56]. Although we approached three women-only services, staff felt there were no eligible residents. As we only interviewed one woman, and three non-white older people we have not been able to focus upon their intersectional experiences within our analysis. However, we were struck in their accounts by the common experiences of feeling unsafe and vulnerable in hostels often related to their age and memory impairment relative to younger residents. There is a need for further research involving older women experiencing homelessness and memory problems as well as those from minoritized and migrant communities. This research utilised a cross sectional design, conducting in-depth interviews at one time point to explore individual perspectives, while our findings show the need to take a more relational perspective and methods that capture complex social interactions over time. Additionally, the background information we collected all relied on self-reporting as we were using the information to contextualise our analysis rather than for quantitative measurement. Participant observation of older hostel residents with memory problems and staff is ongoing, and our team is conducting a longitudinal qualitative study with women experiencing homelessness, most of whom are aged over fifty. In the future we will be testing our co-produced intervention in a feasibility trial and will be reporting on quantitative outcomes collected at two time points.

## Conclusions

Our findings build on what is already known. We found that the changing circumstances and the frequently unrecognised difficulties of those living with memory problems and experiencing homelessness increased their vulnerability and disorientation. These findings complement a growing body of epidemiological and quantitative observational studies in the field, contributing in-depth rich data on lived experiences and presenting indicative directions for staff intervention and improvements in care and support for this population.

### Electronic supplementary material

Below is the link to the electronic supplementary material.

Supplementary Material 1

Supplementary Material 2

## Data Availability

The qualitative data used and analysed during the current study are available from the corresponding author on reasonable request.

## Abbreviations

ARBD: Alcohol Related Brain Damage
CQC: Care Quality Commission
HSCP: Health and Social Care Practitioner
LA: Local Authority
NHS: National Health Service
TBI: Traumatic Brain Injury
