# Supplementary material for: A qualitative exploration of older people’s lived experiences of homelessness and memory problems – stakeholder perspectives
Source: BMC Geriatr. 2023 Sep 12;23:556. doi: 10.1186/s12877-023-04250-0 (PMC10498566; doi:10.1186/s12877-023-04250-0)
Supplement: Supplementary file 1 — Supplementary Material 1 [file 12877_2023_4250_MOESM1_ESM.docx]

Appendix A: Semi-structured interview schedules.

**Interview topic guide for hostel staff and managers**

Thank you for agreeing to talk with me. This part of our study is to help us understand better how to support people who are experiencing homelessness and living with memory problems.

In order to make sure that I don’t miss anything, I will record our conversation on a digital recorder and then it will be professionally transcribed. Whilst we are talking try not to use anyone’s names or information which may identify them. Once the interview has been transcribed, I will ensure that all identifying characteristics are removed so that you, your organisation and anyone you talk about cannot be identified. Once again, everything you tell me will be treated with complete confidence unless you indicate that you or someone else may be at risk of serious harm. If at any point during the interview you feel that you need to stop or leave the room please do tell me. Do you have any questions?

**Interview**

**To start, could you tell me a little about your work? How are you involved with caring for people experiencing homelessness and or memory problems?**

- Can you describe (without using name) a particular older person who you have worked with and what their difficulties were?
- How did you make sense of and understand their difficulties?
- How did supporting them differ from caring for residents without memory problems?
- How did their memory difficulties impact upon their day-to-day life and wellbeing?
- How did their memory difficulties get in their way of moving to or being in settled accommodation?
- Who else (if anyone) was involved in their care and support from outside the hostel team?

**What do you see as the main challenges in supporting older people with memory problems who are experiencing homelessness and what might help?**

(Prompt in terms of accessing support from outside, offering care and support within the hostel, supporting people to move on from the hostel)

- Challenges / solutions at a service level?
- Challenges / solutions at a team level?
- Challenges / solutions at an individual staff level?
- Challenges related to the individual with memory problems?

**What do you think ‘best practice’ would look like in supporting older people with memory problems who are experiencing homelessness in your hostel?**

- What would good health care look like? (memory assessment, primary care)
- What would good social care look like? (personal care needs, accessing in hostel home care)
- What would help people to move on from homelessness / hostel / temporary accommodation? (where do people with memory problems typically move on to)

**What do you think would be important positive outcomes for older people with memory problems who are experiencing homelessness?**

- What might this look like in practice?
- What / who might help them to achieve these outcomes?
- What factors might get in the way of these positive outcomes?

**We want to develop a support intervention for hostel staff supporting older residents with memory problems. What do you think it would be important to include?**

- What areas or topics would it be important to address? (e.g. recognising memory problems, referral pathways, communication, understanding challenging behaviours, managing risky behaviours)
- Is there any specific training that would be useful for you/your team?
- What may help/make it harder for you/your team to put support strategies and new learning into practice?
- In addition to training what additional support would be useful for you/your team?

**What knowledge and skills might frontline staff in homelessness organisations need to support older people experiencing memory problems?**

- What could help them to build these skills?
- What have you learnt from approaches in other related areas (e.g. dementia care, support in homelessness, drug and alcohol support etc)

**Thank you - Is there anything else you would like to add?**

**Interview topic guide for people with memory problems**

Thank you for agreeing to talk with me. This part of our study is to help us understand better how to support people who are experiencing homelessness and living with memory problems.

To make sure that I don’t miss anything, I will record our conversation on a digital recorder and then it will be typed up. Whilst we are talking try not to use anyone’s names. I will take out any named or places after it is typed to make sure you cannot be identified. Once again, everything you tell me will be treated with complete confidence unless you indicate that you or someone else may be at risk of serious harm.

If at any point during the interview you feel that you need to stop or leave the room please do tell me. Do you have any questions?

**Interview**

**To start, could you tell me a little about yourself? Have you noticed any changes in your memory as you have got older?**

- What changes have you noticed?
- How does it affect what you can do (prompt re functioning, taking care of self, managing money, cooking, cleaning, seeing people, washing).
- What do you think may have caused these problems or may make them worse?
- Have you had any help or support with your memory problems?

**How do you like to spend your time during the day?**

- What is important to you?
- What are your hopes for the future?
- What makes it harder for you to do what you would like?

**How do your memory problems get in the way of doing what you would like?**

- Did / do your memory problems make it harder to move on from a hostel?

**While living in a hostel/ experiencing homelessness what support was/is most helpful?**

(Prompt in terms of accessing support from outside, offering care and support within the hostel, supporting people to move on from the hostel)

- Did you need any extra or special help because of your memory problems, and what was this?
- What helped you to move on (if relevant)

**While living in a hostel/ experiencing homelessness what support was/is not helpful?**

- Was anything missing?
- What didn’t you like?

**We want to develop a support intervention to help hostel staff supporting older residents with memory problems. What do you think it would be important to include?**

- What would you tell hostel staff about what is important to you?
- What advice would you give them?

**Thank you - Is there anything else you would like to add?**

**Interview topic guide for health and social care staff**

Thank you for agreeing to talk with me. This part of our study is to help us understand better how to support people who are experiencing homelessness and living with memory problems.

In order to make sure that I don’t miss anything, I will record our conversation on a digital recorder and then it will be professionally transcribed. Whilst we are talking try not to use anyone’s names or information which may identify them. Once the interview has been transcribed, I will ensure that all identifying characteristics are removed so that you, your organisation and anyone you talk about cannot be identified. Once again, everything you tell me will be treated with complete confidence unless you indicate that you or someone else may be at risk of serious harm. If at any point during the interview you feel that you need to stop or leave the room please do tell me. Do you have any questions?

**Interview**

**To start, could you tell me a little about your work? How are you involved with caring for people experiencing homelessness and or memory problems?**

- Can you describe (without using name) a particular older person who you have worked with and what their difficulties were?
- How did you make sense of and understand their difficulties?
- How did their memory difficulties impact upon their day to day life and wellbeing?
- How did their memory difficulties get in their way of moving to or being in settled accommodation?

**What do you see as the main challenges in supporting older people with memory problems who are experiencing homelessness and what might help?**

- Challenges at a service level?
- Challenges at a team level?
- Challenges at an individual staff level?
- Challenges related to the individual with memory problems?

**What do you think ‘best practice’ would look like in supporting older people with memory problems who are experiencing homelessness?**

- What would good health care look like?
- What would good social care look like?
- What would help people to move on from homelessness / hostel / temporary accommodation?

**What do you think would be important positive outcomes for older people with memory problems who are experiencing homelessness?**

- What might this look like in practice?
- What / who might help them to achieve these outcomes?
- What factors might get in the way of these positive outcomes?

**We want to develop a support intervention for hostel staff supporting older residents with memory problems. What do you think it would be important to include?**

- What areas or topics would it be important to address?
- Is there any specific training that would be useful?
- What may help/make it harder for frontline staff to put support strategies and new learning into practice?

**What knowledge and skills might frontline staff in homelessness organisations need to support older people experiencing memory problems?**

- What could help them to build these skills?
- What could we learn from approaches in other related areas (e.g. dementia care, support in homelessness, drug and alcohol support etc)

**Thank you - Is there anything else you would like to add?**
